# Zinc (Zn) Doping by Hydrothermal and Alkaline Heat-Treatment Methods on Titania Nanotube Arrays for Enhanced Antibacterial Activity

**DOI:** 10.3390/nano13101606

**Published:** 2023-05-10

**Authors:** Abhishek Bhattacharjee, Emma Goodall, Bruno Leandro Pereira, Paulo Soares, Ketul C. Popat

**Affiliations:** 1 School of Advanced Materials Discovery, Colorado State University, Fort Collins, CO 80523, USA; abhishek.bhattacharjee@colostate.edu; 2 School of Biomedical Engineering, Colorado State University, Fort Collins, CO 80523, USA; emma.goodall@colostate.edu; 3 Department of Mechanical Engineering, Pontifícia Universidade Católica do Paraná, Curitiba 80215-901, PR, Brazil; brnlp7@gmail.com (B.L.P.); pa.soares@pucpr.br (P.S.); 4 Department of Mechanical Engineering, Colorado State University, Fort Collins, CO 80523, USA

**Keywords:** titanium, orthopedic implant, zinc doping, antibacterial activity, cytotoxicity, biocompatibility

## Abstract

Titanium (Ti) is a popular biomaterial for orthopedic implant applications due to its superior mechanical properties such as corrosion resistance and low modulus of elasticity. However, around 10% of these implants fail annually due to bacterial infection and poor osseointegration, resulting in severe pain and suffering for the patients. To improve their performance, nanoscale surface modification approaches and doping of trace elements on the surfaces can be utilized which may help in improving cell adhesion for better osseointegration while reducing bacterial infection. In this work, at first, titania (TiO_2_) nanotube arrays (NT) were fabricated on commercially available pure Ti surfaces via anodization. Then zinc (Zn) doping was conducted following two distinct methods: hydrothermal and alkaline heat treatment. Scanning electron microscopic (SEM) images of the prepared surfaces revealed unique surface morphologies, while energy dispersive X-ray spectroscopy (EDS) revealed Zn distribution on the surfaces. Contact angle measurements indicated that NT surfaces were superhydrophilic. X-ray photoelectron spectroscopy (XPS) provided the relative amount of Zn on the surfaces and indicated that hydrothermally treated surfaces had more Zn compared to the alkaline heat-treated surfaces. X-ray crystallography (XRD) and nanoindentation techniques provided the crystal structure and mechanical properties of the surfaces. While testing with adipose-derived stem cells (ADSC), the surfaces showed no apparent cytotoxicity to the cells. Finally, bacteria adhesion and morphology were evaluated on the surfaces after 6 h and 24 h of incubation. From the results, it was confirmed that NT surfaces doped with Zn drastically reduced bacteria adhesion compared to the Ti control. Zn-doped NT surfaces thus offer a potential platform for orthopedic implant application.

## 1. Introduction

Every year, millions of people undergo surgeries that incorporate different orthopedic implants due to bone-related injuries or diseases such as rheumatoid arthritis and osteosarcoma. For these patients, undergoing implant surgery is one of the crucial treatment choices to maintain their quality of life [1]. Although implants can be made of metal, ceramic, polymer, or composite, around 70–80% of all implant materials are made from cobalt-chromium (CoCr) alloys, stainless steel (SS), and titanium (Ti) alloys [2]. Compared to the CoCr alloys and SS, Ti alloys are reported to be advantageous due to improved corrosion resistance, low modulus of elasticity, and biocompatibility [3]. Therefore, a huge amount of (around 1000 tons) Ti alloy implants are inserted in patients annually around the world [2]. However, around 10% of these implants fail each year resulting in revision surgeries and expensive medications throughout the rest of the patient’s lifetime [4]. One of the major reasons for orthopedic implant failures is a pathogenic infection caused by bacteria resulting in osteomyelitis. Bacteria can get attached to the implant surfaces and once in the body can proliferate, leading to biofilm formation and the subsequent failure of the implant [5]. Osteomyelitis can lead to devastating consequences for the patients, such as sepsis and prolonged hospitalization [6]. Along with osteomyelitis, another major complication of orthopedic implants is poor osseointegration [4,7,8]. Osseointegration deals with the anchorage of the implant by forming bone tissue around it without growing any fibrous tissue [9]. Successful osseointegration deals with many facts, including the biocompatibility, macroscopic, and microscopic surface properties of the implant along with the undisturbed healing process of the bone [10]. In the USA, around 1.2 million orthopedic implants are inserted in patients annually, of which 112,000 are projected to fail [11]. Complications and failures related to the implants also create a huge economic toll on society. It was estimated that implant-related medical costs climb up to $3 billion annually in the USA [7]. With an increasingly mobile human population, the number of bone-related injuries will increase along with the number of patients having orthopedic implants. This can result in a higher amount of implant failures. The economic burden and human suffering from pain related to complicated treatment procedures and repeated surgeries will also be the result of such a high number of implant failures.

There has been a lot of research into improving osseointegration and reducing bacteria adhesion on implant surfaces. The two major approaches for reducing bacterial adhesion while improving cell adhesion comprise surface coatings and surface modifications [12]. Modifications of implant surfaces such as the introduction of nanoscale surface characteristics have been reported to be effective in osteogenic cellular interaction and reducing bacterial adhesion on the surfaces. One popular approach to surface modification is a combination of grid-blasting and acid-etching. Pits and spike-like nanostructures are created on the surface by this modification technique that showed improved osseointegration [13,14,15,16,17]. Other methods of surface modification include chemical vapor deposition, sol-gel coatings, hydrothermal treatment, glow discharge plasma treatment, and ion implantation [18,19]. The major focus of these Ti surface modifications is to improve osseointegration to help in orthopedic disease treatment by Ti-based implants. It has been reported that increased hydrophilicity of the surfaces also helps in early osseointegration [20]. One of the quick, convenient, inexpensive, and highly tunable methods of preparing a super-hydrophilic Ti surface is by producing TiO_2_ nanotube arrays (NT) through anodization [19]. NT surfaces have shown cell adhesion to the implant surfaces that results in improved osseointegration [13,21,22,23]. Interestingly, NT surfaces also exhibit improved antibacterial activity [12,24]. This is a useful characteristic of the NT surfaces because they can promote osseointegration while reducing bacterial adhesion. This can provide the necessary biocompatibility to the orthopedic implant. Another interesting fact about NT surfaces is that their tubular structures can be used as a delivery mechanism for antibiotics [25], antibacterial substances [26], and trace elements that are useful for cell growth and proliferation [7].

Trace elements such as silver (Ag) nanoparticles, copper (Cu), strontium (Sr), and zinc (Zn) are critical for cell growth and proliferation around an implant surface that can improve osseointegration. Furthermore, they can also hinder bacterial infection on the implant surfaces. For that reason, the incorporation of trace elements on implant surfaces is gaining popularity. Additionally, metal ion release can be a viable technique to improve the antibacterial property of biomaterials [27]. Among the trace elements, Zn is interesting because it can provide the required cell signaling for improved osseointegration while hindering bacterial adhesion on the implant surface [7]. Zn helps in bone regeneration through osteoblast activity such as osteoblast proliferation, mineralization, and osteoblast marker gene expression [7]. Zn also inhibits bone resorption by reducing osteoclast formation as well as helps in anti-inflammation [7]. Of these interesting characteristics, Zn has gained popularity in preparing biomaterials. Recent work utilized several techniques to dope Zn on Ti surfaces. These techniques involved hydrothermal treatment [7,8,28,29], alkaline heat treatment [30,31], and plasma electrolytic oxidation (PEO) [32,33]. Even though Zn incorporation on implant surfaces can potentially improve the biocompatibility of orthopedic implants, only a few investigations have been reported in the literature. Therefore, examining the trace element incorporation on orthopedic implant surfaces along with their antibacterial and toxicological behavior analysis is imperative.

In this work, Zn doping was conducted on Ti and NT surfaces using two different methods: hydrothermal and alkaline heat treatment. These two processes were selected because they are relatively simple and highly tunable. For the hydrothermal method, the surfaces were incubated with zinc acetate. For the alkaline heat treatment, the surfaces were incubated with a solution containing zinc nitrate hexahydrate and sodium hydroxide. The difference between these two methods was mainly in treatment temperature and time. The hydrothermal method used 200 °C and a 1 h treatment time, whereas the alkaline heat treatment was conducted for 24 h at 60 °C. All the treated surfaces were annealed at 530 °C to crystallize the Zn and NTs. Scanning electron microscopy (SEM) and energy-dispersive X-ray spectroscopy (EDS) were used to understand the morphology, topography, and Zn distribution on the surfaces. X-ray photoelectron spectroscopy (XPS) and X-ray diffraction (XRD) crystallography were used to evaluate surface chemistry and crystalline structure. XPS was also used to quantify Zn on the surfaces. Cytotoxic behavior was analyzed by incubating the surfaces with adipose-derived human stem cells (ADSCs) and examining the amount of lactate dehydrogenase (LDH) released from the cells when in contact with the surfaces. Finally, the antibacterial properties of the surfaces were evaluated by incubating the surfaces with Gram-positive *Staphylococcus aureus* and Gram-negative *Pseudomonas aeruginosa*. SEM and live/dead bacteria staining were used to examine bacteria adhesion and morphology on the surfaces. Experimental results indicated that the Zn-doped surfaces exhibited improved biocompatibility by reducing the bacteria adhesion significantly while not being cytotoxic to the ADSCs.

## 2. Materials and Methods

### 2.1. Titania Nanotube (NT) Fabrication

Titania nanotube (NT) surfaces were fabricated from commercially available 0.5 mm thick pure titanium. First, 2 cm × 2 cm Ti samples were cut and polished using silicon carbide sheets. Then, the samples were soaked in acetone for 3 min and sonicated for 10 min to clean any debris from the surface. The samples were further cleaned using soap and isopropanol followed by 10 min sonication each in isopropanol and deionized water (DI). Finally, the cleaned Ti samples were dried inside a hood before subsequent processing. NT were grown on the surfaces by anodization and annealing. An electrolyte cell was used for the anodization process where a cleaned titanium surface was used as the anode and platinum was used as the cathode. Prior to the anodization process, the platinum surfaces were cleaned thoroughly using nitric acid, isopropanol, and DI water. A solution containing 95% diethylene glycol (DEG, Thermo Fisher Scientific Chemicals Inc., Ward Hill, MA, USA), 2% hydrofluoric acid (HF, 48%, KMG Electronic Chemicals, Inc., Houston, TX, USA), and 3% DI water was used as an electrolyte. A titanium anode and a platinum cathode were hooked to the electrolyte cell using alligator tips. Care was taken when hooking the samples so that the alligator tips did not touch the electrolyte solution. Then, 55 V of electricity was supplied for 22 h at room temperature for the anodization process. After the anodization process, the NT surfaces were removed and washed using DI water and isopropanol before drying inside a hood. Finally, the NT surfaces were annealed at 530 °C for 3 h with 15 °C/min temperature increments and stored before being used for subsequent experiments. Cleaned and polished titanium (Ti) was also prepared to be used as a control.

### 2.2. Zinc Doping on Ti and NT Surfaces

Zinc (Zn) doping on Ti and NT surfaces was conducted by two different methods: hydrothermal and alkaline heat treatment. To prepare hydrothermally Zn-doped NT surfaces (NT_hyt_), un-annealed NT surfaces were used. These surfaces were incubated in 0.1 M zinc acetate (ThermoFisher Scientific, Waltham, MA, USA) solution at 200 °C for 1 h inside a polytetrafluoroethylene (PTFE) lined hydrothermal autoclave reactor. The surfaces were taken out from the reactor and thoroughly washed in DI water for 5 min. Finally, the surfaces were annealed similarly as described in Section 2.1 prior to storing for subsequent experiments.

To prepare alkaline heat-treated NT surfaces (NT_alk_), un-annealed NT surfaces were used. These surfaces were incubated in 10.71% zinc nitrate hexahydrate (Oakwood Chemical, Estill, SC, USA), 17.31% sodium hydroxide (Fisher Chemical, Fair Lawn, NJ, USA), and 71.98% DI water solution at 60 °C for 24 h inside a PTFE container. The surfaces were taken out of the reactor and thoroughly washed in DI water for 5 min. Finally, the surfaces were annealed similarly as described in Section 2.1 prior to storing for subsequent experiments.

Ti surfaces were also treated in a similar way to prepare hydrothermally treated Ti_hyt_ and alkaline heat-treated Ti_alk_ surfaces.

### 2.3. Material Characterization

To understand the morphological and topographical characteristics of different surfaces (Ti, Ti_hyt_, Ti_alk_, NT, NT_hyt_, and NT_alk_), a JEOL JSM-6500F field emission scanning electron microscope (FESEM) was used at 15 kV. Scanning electron microscopic (SEM) images were taken from each surface at varying magnifications ranging from 500× to 30,000×. Working distance, brightness, and contrast were optimized for each image to ensure high-quality SEM images of the surfaces were produced. Furthermore, energy-dispersive X-ray spectroscopy (EDS) spectra were collected with an Oxford SDD EDS detector connected to the FESEM. For each surface, element mapping was conducted to understand the elemental distribution on the surfaces. For NT_hyt_, NT_alk_, Ti_hyt_, and Ti_alk_ surfaces, elemental mapping was also conducted in specific zones to understand special features on the surfaces. All EDS data were analyzed using Oxford Aztec software.

To understand the surface wettability, the static contact angle was measured for all the surfaces by using a Ramé-Hart goniometer (Ramé-Hart Instrument Co., Succasunna, NJ, USA). A 10 µL drop of water was placed on each surface using a micrometer syringe. Then static contact angle and image of the droplet on the surfaces were collected using DROPimage software.

To understand the surface chemistry, X-ray photoelectron spectroscopy (XPS) was utilized. An XPS survey was taken with a PHI Physical Electronics PE-5800 X-ray Photoelectron Spectrometer with an Al Kα X-ray source. Survey spectra for all the surfaces were collected from 0 eV to 1100 eV. Peak-fit analysis was conducted using MultiPak (version 9.6.1.7). From the survey spectra, elemental analysis was also conducted for each surface using the MultiPak (version 9.6.1.7) and the composition (atomic weight percentage, %at) of each element was recorded.

The crystalline structure of the surfaces was analyzed by utilizing X-ray diffraction (XRD, XRD-7000 Shimadzu) while using CuKα radiation at 40 kV and 30 mA. When performing XRD, a thin film (TF-XRD) geometry was utilized for the surfaces with a fixed incidence angle of 5°. Diffractograms were acquired with continuous scans from 20° to 80° at a scanning speed of 1°/min. Peaks were indexed using Match! software with the PDF2 database.

### 2.4. Stability of Zn-Doped Surfaces

To understand the stability of the Zn-doped surfaces, elemental analysis from the XPS survey scan was used. First, 2 cm × 2 cm surfaces were collected and incubated in DI water for 4 weeks (28 days) at room temperature. Then the surfaces were dried inside a hood and XPS surveys were taken following the method described in Section 2.3. From these survey scans, %at was calculated for each of the surfaces. Finally, the %at of the elements after 4 weeks was compared with data gathered in Section 2.3 to evaluate the changes in Zn composition after 28 days in DI water.

### 2.5. Mechanical Properties of the Surfaces

To understand the mechanical properties, such as surface hardness and elastic modulus, a nanoindentation technique was utilized. A Nanoindenter (ZwicK-Roell/Asmec) was used to measure the surface hardness (H) and elastic modulus (E). It was programmed by an array (5 × 5), with a distance of 50 µm between each indentation and 0.1 N of maximum applied force on the surface by a calibrated Berkovich tip. The indentation method used was the quasi continuous stiffness measurement (QCSM) method. This method allows for high accuracy measurements due to a progressively increasing force (from 0–100 mN for this study) combined with a dwell time at each force point.

### 2.6. Cytotoxicity Behavior of the Surfaces

To evaluate the cytotoxicity induced by the surfaces, a lactate dehydrogenase (LDH) based indicator assay was utilized (CyQUANT™ LDH Cytotoxicity Assay Kit, ThermoFisher Scientific, Waltham, MA, USA). The cytotoxicity of the surfaces was evaluated with adipose-derived adult stem cells (ADSCs). The ADSCs were cultured in a growth medium containing 90% MEM Alpha Modification (1×, cytiva, Marlborough, MA, USA), 9% fetal bovine serum (FBS), and 1% penicillin-streptomycin. The surfaces were taken into a 48-well plate and sterilized by UV light for 1 h followed by washing with PBS two times. After that, the surfaces were incubated for 24 h at 5% CO_2_ with 20,000 ADSC cells/mL. After the incubation, 50 µL supernatants of the cell media were taken from each surface in a sterile 96-well plate. Then the manufacturer’s protocol was followed to determine the cytotoxicity induced by the surfaces against the ADSCs.

### 2.7. Bacteria Culture

To evaluate the antibacterial properties of different surfaces, Gram-positive *Staphylococcus aureus* (*S. aureus*, ATCC6538) and Gram-negative *Pseudomonas aeruginosa* (*P. aeruginosa*, ATCC10145) bacterial strains were used. Both bacteria were grown in tryptic soy broth (TSB, Sigma-Aldrich, St. Louis, MO, USA) at 37 °C for 24 h until a bacterial concentration of 10^9^ Colony Forming Units (CFU)/mL was achieved. The CFU/mL was measured by determining the absorbance values of bacterial solution using a plate reader at 562 nm wavelength. To understand the bacteria adhesion and morphology on the surfaces, a diluted bacteria culture of 10^6^ CFU/mL was used. The surfaces were taken into a 48-well plate and sterilized by UV light for 30 min and subsequently washed twice with PBS for 5 min. Then the surfaces were incubated with the 10^6^ CFU/mL bacteria solution for 6 h and 24 h at 37 °C inside an incubator. After the incubation, the surfaces were washed twice with PBS for 5 min to remove any non-adhered bacteria from the surfaces before subsequent characterization.

#### 2.7.1. Bacteria Adhesion on Different Surfaces

For evaluating the number of live or dead bacteria adhering on different surfaces, a fluorescence microscope was used. For this, the surfaces were incubated at room temperature for 15 min in a stain solution containing a 1:1 ratio of propidium iodide (dead bacteria stain) and Syto 9 (live bacterial stain) (3 µL/mL in PBS, ThermoFisher Scientific, Waltham, MA, USA). Then, the stain solution was removed, and the surfaces were incubated with 3.7% formaldehyde (Fisher Chemical, Fair Lawn, NJ, USA) for 15 min at room temperature. After that, the formaldehyde was removed, and the surfaces were rinsed with PBS twice for 5 min. Immediately after that, the surfaces were imaged using a fluorescence microscope. ImageJ was used to evaluate the percentage of area fraction covered by the live or dead bacteria on the surfaces. From staining to imaging, all the procedures were conducted in the dark.

#### 2.7.2. Bacteria Morphology on Different Surfaces

To characterize adhered bacteria morphology on different surfaces, SEM images were collected for each surface after incubating with bacteria. After bacteria incubation, the surfaces were incubated with a primary fixative solution containing 3% glutaraldehyde (Sigma-Aldrich, St. Louis, MO, USA), 0.1 M sucrose (Sigma-Aldrich, St. Louis, MO, USA), and 0.1 M sodium cacodylate (Electron Microscopy Sciences, Hatfield, PA, USA) in DI water for 45 min at room temperature. Then the fixative solution was removed, and the surfaces were incubated with a buffer solution containing the fixative solution without the glutaraldehyde for 10 min. Finally, the surfaces were dehydrated using 35%, 50%, 70%, and 100% ethanol solution (10 min incubation in each solution). Before imaging with SEM, the surfaces were kept dry inside a desiccator. Right before loading the surfaces in the SEM instrument, a 10 nm gold coating was added utilizing a Denton Vacuum Desk II Gold Sputter Coater to improve surface conductivity for imaging.

### 2.8. Statistical Analysis

For surface characterization, at least 3 samples (*n_min_* = 3) of each surface were used. To evaluate the cytotoxicity, at least 4 samples (*n_min_* = 4) of each surface were incubated with ADSCs. For antibacterial activity studies, 3 samples from each surface were used and all the experiments were repeated at least twice (*n_min_* = 6). For statistical analysis, a two-way analysis of variance test (ANOVA) was conducted followed by a post-hoc analysis (*t*-test). The results were considered statistically significant when the *p* value was less than 0.05.

## 3. Results and Discussion

### 3.1. Morphology and Zn Distribution on the Surfaces

**Figure 1:**
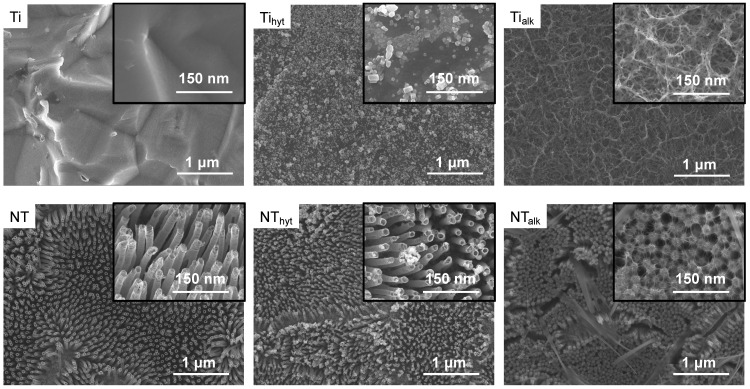
Representative SEM images of different surfaces at low (5000×) and high magnification (insert, 30,000×).

Morphological and topographical features of the Ti and NT surfaces were characterized by SEM images. The morphological features on the surfaces are important for biomaterials since they can affect cell and bacteria adhesion on surfaces. Surface properties, such as roughness, have been reported to improve cell adhesion and osseointegration [34,35]. Titania nanotube array (NT) and Zn incorporation can effectively change the morphological properties of the surfaces due to treatments utilizing different chemicals and temperature ranges. Figure 1 shows the representative SEM images of different surfaces. The Zn-doped Ti_hyt_ and Ti_alk_ surfaces exhibited significant differences in the surface morphology. On the Ti_hyt_ surface, crystal-like structures were found relatively evenly distributed throughout the surface. This is significantly different from the Ti control surface where such crystal-like structures were absent. Thus, it was hypothesized that these crystals were Zn crystals formed due to the hydrothermal process, and this was further confirmed by EDS (discussed later). The Ti_alk_ surface exhibited a web-like structure. This is also significantly different compared to the Ti and Ti_hyt_ surfaces. Alvarez et al. (2009) performed an alkali heat treatment on Ti implants containing [Zn(OH)_4_]^2−^ complex and found a similar web-like structure, which they named a reticulated microporous structure [30]. Manivasagam and Popat (2020) also treated Ti implants with an alkali solution at 60 °C for 24 h and found a similar web-like structure [36]. So, the alkaline heat treatment in this study conformed to the reported literature. From the SEM images collected on NT surfaces, it was evident that the anodization and annealing processes were successful in producing an evenly distributed NT array. The tubular geometrical shapes were distinctly visible on NT surfaces, which resemble the NT shapes described in the previously reported literature [4,37,38]. The roughness of the implant surface is necessary to biomimic the bone microenvironment inside a human body. NT arrays can provide that required roughness, which can help in cell proliferation and viability. Moreover, the tubular-shaped geometry can hinder the growth of bacterial adhesion due to charge repulsion, bacteria membrane stretching by the NTs, and variations in surface roughness [12]. When these NT surfaces were doped with Zn, a similar trend to that of Ti_hyt_ and Ti_alk_ was observed. The NT_hyt_ surfaces were prepared by hydrothermal treatment and had a crystal-like structure on top of the NTs. The Zn crystals were also evenly distributed here but not as visible as the Ti_hyt_ because of the nanostructured surface architecture. Some of the Zn crystals were smaller than the diameter of the NTs and the space between the surrounding NTs. So, it is possible that some crystals filled up the space in these empty channels. For the alkaline heat-treated NT_alk_ surface, a familiar web-like structure was visible. The web was formed on top of the NTs and was evenly distributed throughout the surface. However, a new rod-shaped feature was found on the NT_alk_ surfaces which was absent on the Ti_alk_ surfaces. These features were probably arising from the sodium (Na) content in the alkaline (NaOH) solution used for Zn doping, which was later examined utilizing EDS. From the morphological analysis of the surfaces, it was evident that two Zn doping techniques initiated distinct morphological features on the surfaces compared to the pure Ti and NT controls.

**Figure 2:**
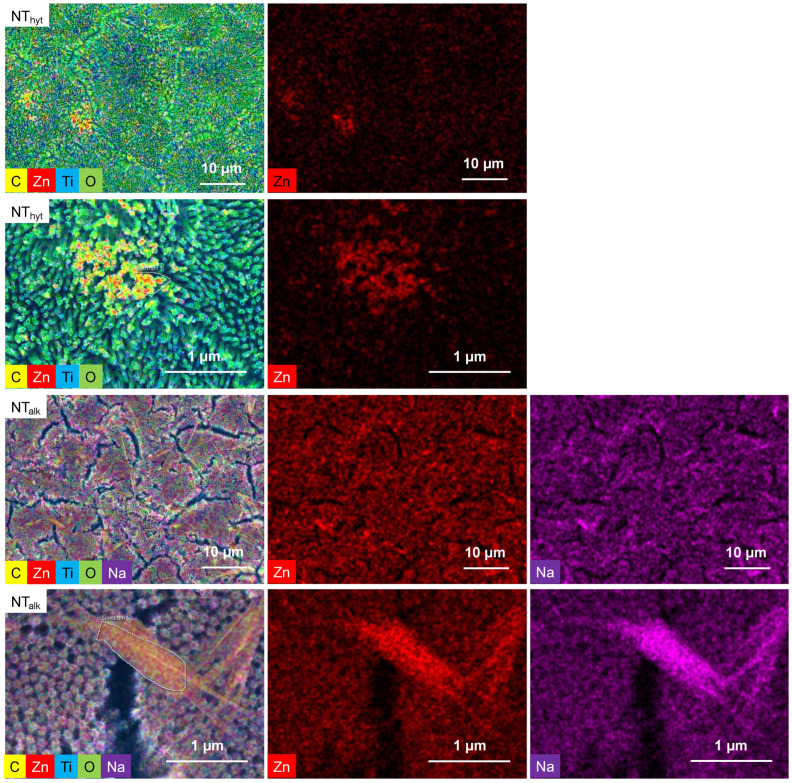
EDS analysis of representative surfaces NT_hyt_ and NT_alk_. The top two rows show the EDS layered images of NT_hyt_ at different magnifications along with the Zn distribution. The bottom two rows show the EDS layered images of NT_alk_ at different magnifications along with Zn and Na distribution. White outlines in NT_hyt_ (2nd row) and NT_alk_ (4th row) is showing the crystal and rod-shaped features on the NT_hyt_ and NT_alk_ surfaces respectively.

To understand the distribution of different elements on the surfaces, EDS mapping was conducted. The EDS mapping was especially helpful for understanding Zn-doping efficiency and the distribution of Zn throughout the surfaces. Figure 2 shows representative EDS maps of NT_hyt_ and NT_alk_ surfaces. For the hydrothermally treated Ti_hyt_ and NT_hyt_ surfaces, although the Zn was evenly distributed, its concentration was much higher where the crystal-like structures were present. Hence, a higher magnification image was taken to understand the elemental composition of these crystal-like structures (Figure 2). The magnified EDS mapping indicated a higher concentration of Zn on these crystal-like structures, thus proving the crystals were indeed Zn crystals. For alkaline heat-treated surfaces (Ti_alk_ and NT_alk_), Zn was found to be evenly distributed. Na distribution was also found for the alkaline heat-treated surfaces that might result from the residual NaOH used in the Zn-doping treatment. However, the distribution of Na from EDS mapping can be misleading since the characteristics X-ray released by these two elements are very similar in energy level (Na, Kα = 1.04 eV and Zn, Lα = 1.012). Yet, the EDS spectra signaled the presence of Na on these surfaces, which was later confirmed utilizing XPS. A rod-shaped feature was found to be present on the NT_alk_ surface. High-magnification mapping was performed for these interesting features, which is shown in Figure 2. From the EDS mapping, it was found that these features mainly consisted of Zn, Na, and O. So, from the EDS mapping, it was understood that the web-like structure was mainly formed by the alkaline heat treatment of Zn and the rod-like features were formed by the Zn and excess Na on the surfaces. Na was also found to be present in Ti_alk_ surfaces; although, they did not create a rod-like feature on the surface. This interesting difference can result from the presence of NT arrays and their interaction with an alkaline solution during the alkaline heat treatment process.

### 3.2. Surface Chemistry

**Figure 3:**
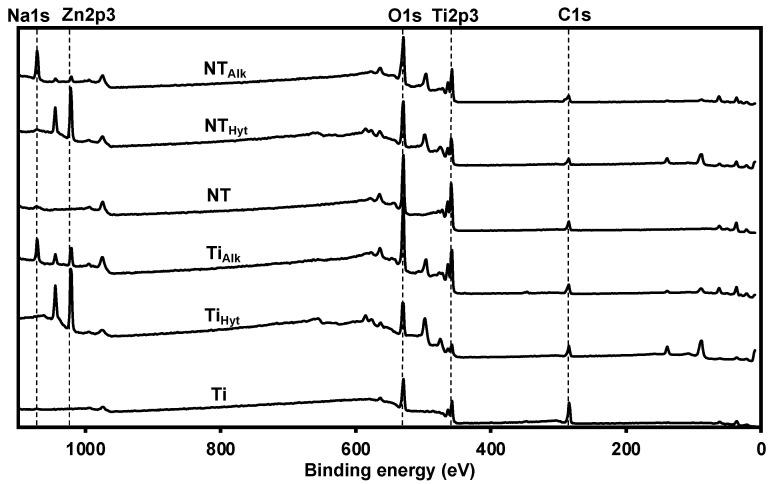
XPS survey spectra of different surfaces.

The pure Ti surface underwent multiple surface modifications in this work to improve its biocompatibility. Hence, it is important to understand the chemical properties of the surfaces before and after these modifications. Additionally, evaluating the surface chemistry is required to understand if the Zn doping was successful or not. As a first step, XPS survey scans were collected for all the surfaces. XPS survey scans can identify the elements present on the surfaces along with their relative concentration. From the survey scan of the Ti control, the presence of Ti2p3, C1s, and O1s was detected, which is typical of the Ti XPS spectrum reported in the previous literature [38]. However, after the Zn doping, both the Ti_hyt_ and Ti_alk_ exhibited a new peak at binding energy 1022 eV that corresponds to the presence of Zn2p3 on the surfaces (Figure 3). The prominence of this Zn2p3 peak was less in Ti_alk_ compared to the Ti_hyt_, which indicated a lower amount of Zn presence on the alkaline-treated surfaces. However, Ti_alk_ exhibited another peak at binding energy 1071.4 eV corresponding to Na1s, which came from the NaOH used in the solution for alkaline heat treatment. The NT surface spectrum was similar to the Ti control, except for the high prominence of the O1s peak (530 eV) due to the presence of titania (TiO_2_) on the surface. Like the Ti_hyt_ and Ti_alk_, the Zn-doped NT_hyt_ and NT_alk_ surfaces also showed a new Zn2p3 peak (1022 eV), which was absent both in the Ti and NT surfaces, thus confirming the successful doping of Zn on the surfaces. Similar to the Ti_alk_, the NT_alk_ also exhibited an Na peak (1071.4 eV) due to the presence of NaOH in an alkaline solution.

From the XPS survey scans, the elemental composition (atomic weight percentage, %at) of the surfaces was calculated (Table 1). Both Ti and NT showed no Zn or Na as expected. The %at of O increased from 34.7% to 57.3% between Ti and NT, indicating the presence of TiO_2_ on the surface. Both the hydrothermally treated surfaces (Ti_hyt_ and NT_hyt_) showed a higher amount of Zn compared to the alkaline heat-treated surfaces. The amount of Zn on Ti_hyt_ was higher (21.6%) compared to NT_hyt_ (16.9%), possibly due to the presence of a higher amount of O on NT_hyt_. On the other hand, both alkaline heat-treated surfaces showed Na along with Zn. The amount of Na was higher in NT_alk_ (18.1%) compared to Ti_alk_ (9.8%), which probably affected the relative percentage of Zn (NT_alk_ 1.3% and Ti_alk_ 4.1%) on these surfaces.

### 3.3. Surface Crystallinity

**Figure 4:**
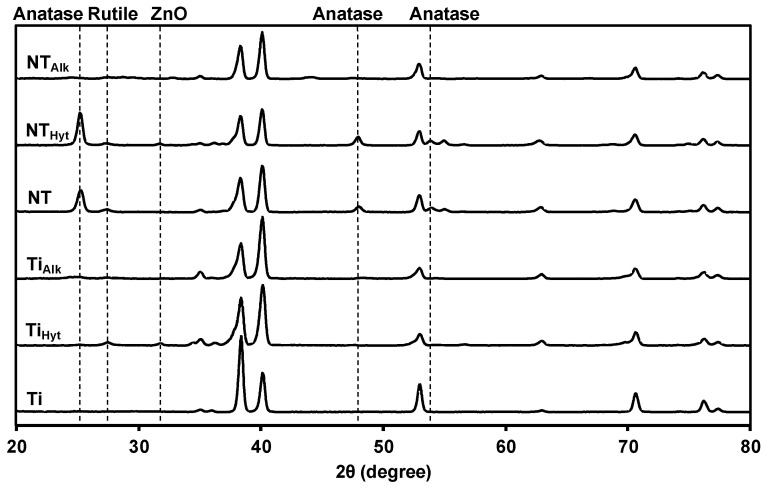
XRD spectra of different surfaces.

Both the NT array preparation and Zn-doping techniques used higher temperatures, which can alter the crystalline phases of the surfaces. So, identifying these phases is important for this study. After anodization, NT arrays were amorphous, but after the annealing treatment at 530 °C, stable rutile and anatase crystalline structures were formed on the NT surfaces [35]. It is reported that the presence of rutile and anatase phases on the implant surface can help in osteogenic activity and osseointegration [39,40,41]. NT surfaces containing anatase and rutile phases also have been reported to possess antibacterial properties by reducing antibacterial adhesion on the surfaces [42,43,44]. In this work, all the NT surfaces showed a mixture of rutile and anatase phases (Figure 4). NT and NT_hyt_ surfaces had prominent anatase (25.27°, 48.01°, 53.91°) and rutile peaks (27.43°); however, these peaks were not as prominent in NT_alk_, possibly due to the presence of web-like structure on top of the TiO_2_ nanotubes. On the other hand, Ti control did not have any of these anatase or rutile phases. Ti_hyt_ and Ti_alk_ showed the stable rutile phase (27.43°) coming from the high-temperature treatment for Zn doping. All the Zn-doped surfaces also showed a ZnO peak (31.73°), which confirmed the presence of Zn on the surfaces once again. Similar to the other peaks, the alkaline heat-treated surfaces showed less prominence of ZnO peak possibly due to the lower amount of Zn on the surfaces. Unmarked peaks in Figure 4 correspond to Ti.

### 3.4. Surface Stability

Surface stability is a key factor for biomaterials. Since implants are intended to stay inside the human body for a long period of time, it is important to understand the stability of the Zn-doped surfaces. The stability of the Zn-doped surfaces was evaluated up to 4 weeks (28 days) of incubation in DI water by taking an XPS survey scan and calculating surface elemental compositions (%at). Table 2 shows the %at of Zn and Na before and after 28 days of incubation in DI water. After 4 weeks, there was a significant decrease in Zn for the hydrothermally treated surfaces Ti_hyt_ and NT_hyt_. Zn content in the Ti_hyt_ and NT_hyt_ reduced by almost 59% and 45%, respectively. Whereas the alkaline heat-treated samples Ti_alk_ and NT_alk_ showed a significant increase in Zn. However, this is not due to an increase in the Zn content on the surfaces, but rather a decrease in the Na content, which resulted in an increase in the relative %at of Zn after 28 days. Since the surfaces were incubated in DI water, the Na might have been dissolved in water resulting in a significant decrease of Na on the surfaces after 28 days. The Ti control and NT surfaces did not show any change during this experiment.

### 3.5. Surface Wettability

**Figure 5:**
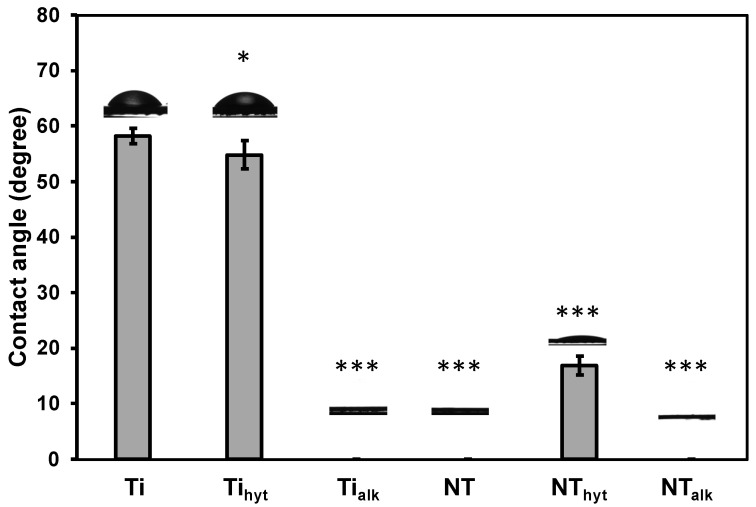
Static water contact angle of different surfaces. Images of water droplet on the surfaces are also shown in the plot. Statistical significances (*p*-value) were represented as * *p* < 0.05 and *** *p* < 0.001.

Surface wettability is another important surface characteristic of an implant’s biocompatibility. Hydrophilic surfaces have been reported to promote cell adhesion and proliferation [45]. Hydrophilic surfaces can also reduce bacterial adhesion [43]. Surface wettability can be calculated by measuring the apparent static contact angle using a droplet of water on the surface. When the contact angle (θ) is lower than 90°, the surfaces are defined as hydrophilic, and when $\theta <10^{\circ }$, then the surfaces are defined as superhydrophilic. All the treated surfaces in this work exhibited a significantly reduced contact angle compared to the Ti control. Ti, Ti_hyt_, and NT_hyt_ were hydrophilic surfaces, whereas Ti_alk_, NT, and NT_alk_ surfaces were superhydrophilic (Figure 5). All the surfaces except the Ti control were heat treated during annealing at 530 °C. During this heat treatment, the hydrophilic property of the surfaces increased, which matched the reported literature [43]. Thus, all the Zn-doped surfaces should potentially reduce bacterial adhesion and improve cell proliferation on the implant surfaces due to their superhydrophilic nature.

### 3.6. Mechanical Properties

Mechanical properties such as material hardness, stiffness, and flexibility are important factors that determine cell-material interactions and ultimately cell fate on implant surfaces [46]. Several studies have reported that cells receive mechanical signaling from the surrounding microenvironment and prefer softer surfaces that promote cell adhesion on the material [46,47,48,49]. NT surfaces have nanoscale topographical features that can provide less restriction to deformation, resulting in a softer surface compared to an unmodified Ti surface [48]. To understand these mechanical properties, indentation hardness and elastic modulus of all the surfaces were evaluated using the nanoindentation technique. Table 3 shows the hardness and elastic modulus values of different surfaces. NT and NT_hyt_ showed the minimum hardness values of 0.32 ± 0.02 GPa and 0.26 ± 0.02 Gpa, respectively, whereas Ti control had a hardness value of 2.3 ± 0.4 GPa. The NT_alk_ exhibited a higher hardness value of 1.00 ± 0.2 GPa, which was higher than other NT surfaces, probably due to the presence of a web-like structure on top of the nanotubes as discussed in the morphological analysis. This web-like structure created a separate layer on the nanotubes and resulted in a higher restriction to deformation, which ultimately increased the hardness value. A similar result was observed for the Ti_alk_, which exhibited a similar hardness value of 2.4 ± 0.2 GPa. Elastic modulus values also showed a similar trend to the hardness values (Table 3). All the NT surfaces exhibited lower elastic modulus compared to the Ti surfaces. This is an expected result due to the topographical differences of the Ti and NT surfaces that changed the restriction towards nanoindenter force.

### 3.7. Cytotoxicity of the Surfaces

**Figure 6:**
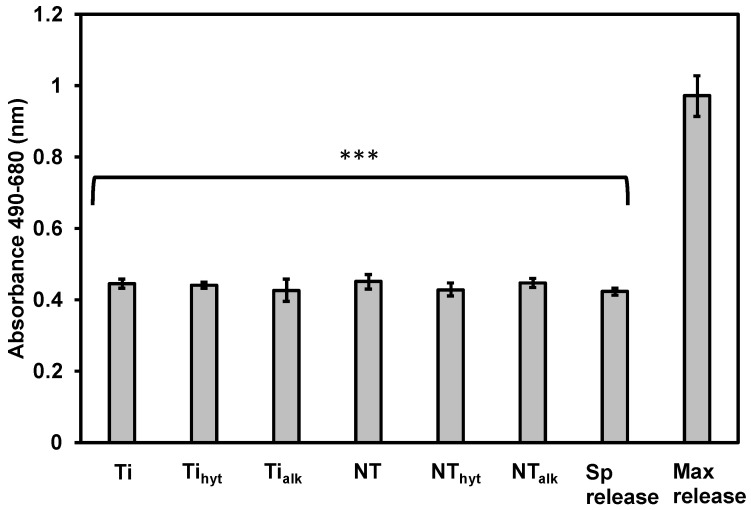
Cytotoxicity study of the surfaces against ADSCs. The values are represented as absorbance values at 490 and 680 nm. Sp release and Max release represent spontaneous and maximum LDH release values. *** represents *p* value < 0.001 when compared to the max release.

Cytotoxicity of different surfaces was determined using adipose-derived stem cells (ADSCs). The surfaces were incubated with ADSCs for 24 h before the lactate dehydrogenase (LDH) released by the damaged cells was calculated. A higher amount of LDH indicates a highly cytotoxic surface. The maximum release of LDH was calculated where all the cells were intentionally damaged to get the highest amount of LDH. A spontaneous release value was also calculated where no cells were damaged. When compared to these controls, all the surfaces showed no apparent cytotoxicity (Figure 6).

### 3.8. Bacteria Adhesion

**Figure 7:**
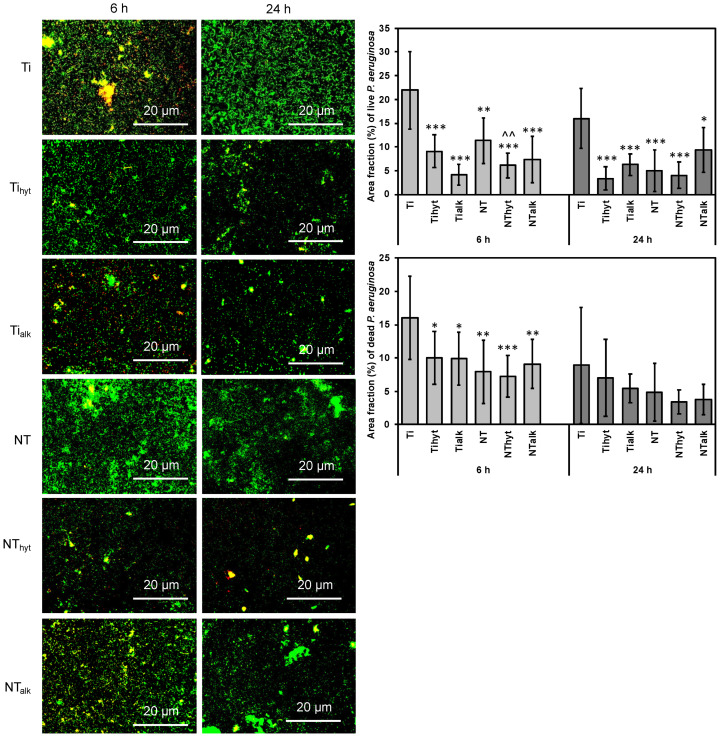
Representative fluorescence microscopic images of surfaces after incubation with *P. aeruginosa*. The graphs represent the number of live and dead *P. aeruginosa* attached to the surfaces. *, **, *** represent *p*-value < 0.05, <0.01, and <0.001 when compared with control Ti. ^^ represents *p* value < 0.01 when compared with NT.

Bacterial infection is one of the major causes of orthopedic implant failure. Bacteria can attach to the implant surface and start to proliferate, forming biofilms that protect the bacteria colony from the surrounding immune response [12]. Most of the implant-related infections is caused mainly by the Gram-positive bacteria genus of *Staphylococcus* with *Staphylococcus aureus* (*S. aureus*) being a prominent infection-creating pathogen in this bacteria family [50,51,52]. Arciola et al. (2005) surveyed 1027 isolates from 699 patients undergoing revision orthopedic surgeries and found the majority of the infection was caused by *S. aureus* (35.5%) [50]. The second most prominent genus was Gram-negative *Pseudomonas*, with *Pseudomonas aeruginosa* (*P. aeruginosa*) being the most prominent infection-creating pathogen in this genus (6.7%) [50]. Even after maintaining a highly sterile environment during orthopedic implant surgeries, bacteria can still proliferate inside the host body [53]. Therefore, implants having antibacterial characteristics are a crucial step to reduce implant-related bacterial infection. To assess the antibacterial activity of the Zn-doped surfaces, all the surfaces were incubated for 6 h and 24 h with *P. aeruginosa* and *S. aureus*. Then live/dead bacteria staining was performed to evaluate the number of bacteria attached to each surface. Figure 7 shows the fluorescence microscopic images of the surfaces after the incubation periods with *P. aeruginosa*. Figure 7 also shows the two plots that quantified live and dead *P. aeruginosa* attached to different surfaces. Ti had the most bacteria attached to the surface for both 6 h and 24 h incubation periods. The Zn-doped Ti_hyt_ and Ti_alk_ had significantly lower *P. aeruginosa* attached to their surfaces. NT had lower *P. aeruginosa* attached to its surface when compared to the control Ti. All the Zn-doped surfaces reduced the *P. aeruginosa* attachment; however, the least reduction of bacteria attachment came from the NT_hyt_ surface. For instance, the area fraction % of Ti control by live *P. aeruginosa* after 6 h of incubation was 21.94%, whereas NT_hyt_ was 6.1% (Figure 7). The hydrothermally treated NT_hyt_ surface had more Zn compared to the NT_alk_. So, the number of bacteria attached to the NT_hyt_ was less than NT_alk_.

**Figure 8:**
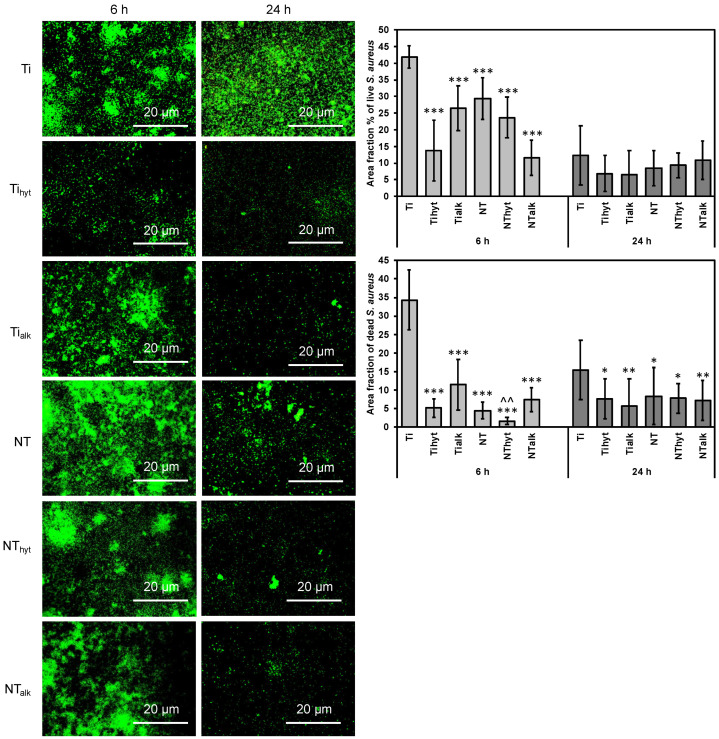
Representative fluorescence microscopic images of surfaces after incubation with *S. aureus*. The graphs represent the number of live and dead *S. aureus* attached to the surfaces. *, **, *** represent *p*-value < 0.05, <0.01, and <0.001 when compared with control Ti. ^^ represents *p* value < 0.01 when compared with NT.

Similar to *P. aeruginosa*, *S. aureus* attachment was also found to be low in Zn-doped surfaces compared to the control Ti. Figure 8 shows the fluorescence microscopic images and quantified plots for *S. aureus* adhesion on the surfaces. After 6 h of incubation, both dead and live *S. aureus* adhesion were significantly reduced for Zn-doped Ti and NT surfaces compared to the Ti control. For instance, the area fraction % of Ti control by live *S. aureus* after 6 h of incubation was 41.95%, whereas NT_hyt_ and NT_alk_ were 23.73% and 11.58%, respectively (Figure 8). After 24 h of incubation, dead *S. aureus* reduction was also significantly reduced in the Zn-doped surfaces. Only in one case did the Ti control have better performance for live *S. aureus* reduction, which was after 24 h of incubation. All the Zn-doped surfaces also reduced *S. aureus* adhesion during this time; however, they were not significantly different from the Ti control.

### 3.9. Bacteria Morphology

**Figure 9:**
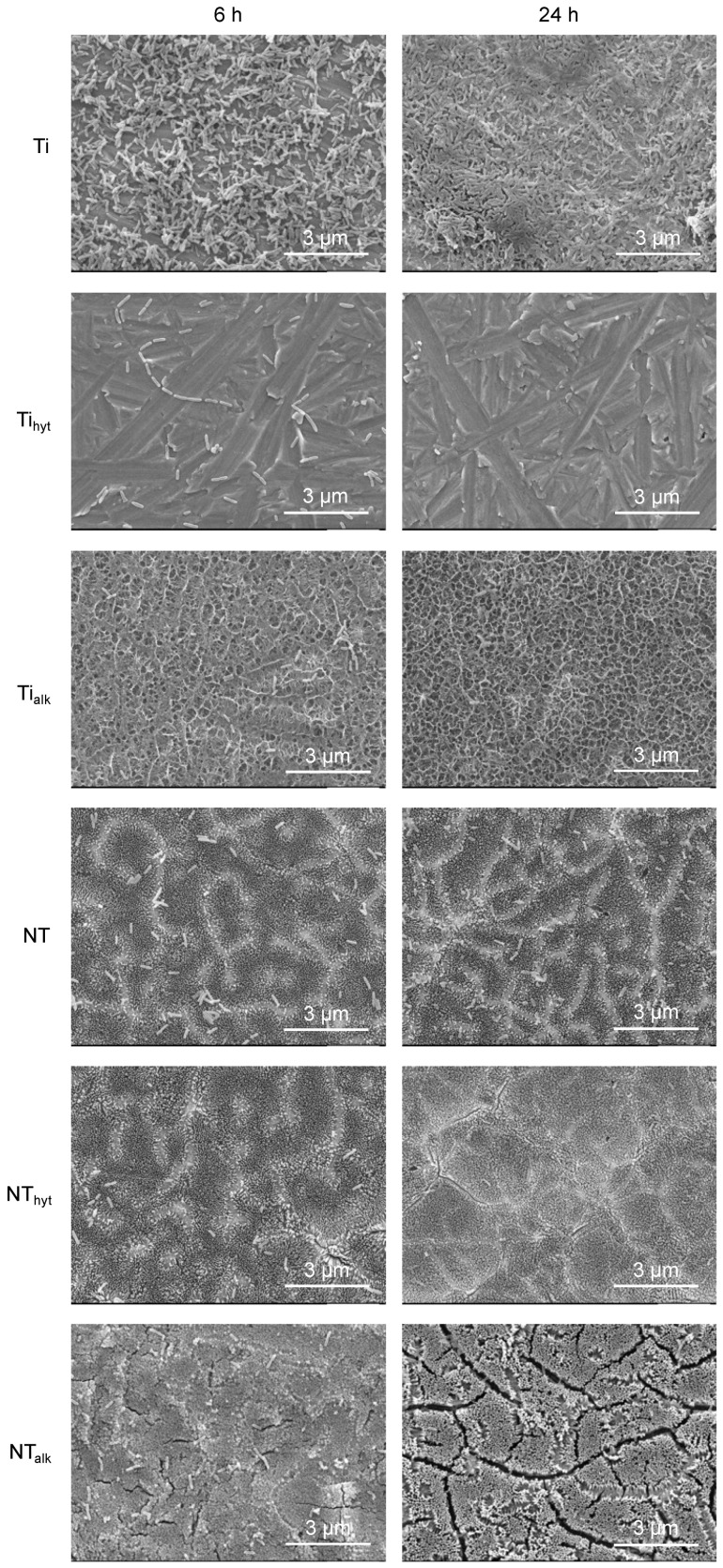
Representative SEM images of bacteria morphology after 6 h and 24 h of *P. aeruginosa* incubation.

Morphological analysis of viable bacteria on the implant surfaces is important to understand how bacteria is attaching, proliferating, and forming biofilms. If bacteria can form a biofilm on the surface, it can protect the bacteria from antibacterial agents and can proliferate until the implant fails. So, to understand bacteria morphology on the surfaces, SEM images were taken after the bacteria were fixed by using relevant adhesives. Figure 9 shows the SEM images of the surfaces after 6 h and 24 h of *P. aeruginosa* incubation. Similar to the results described earlier, the Zn-doped surfaces reduced the *P. aeruginosa* attachment drastically. NT also reduced the *P. aeruginosa* on its surface; however, most reduction came from the NT_hyt_ and Ti_hyt_. Both surfaces contained a higher amount of Zn compared to the NT_alk_ and Ti_alk_, and hence, their antibacterial activity was improved. After 24 h of incubation, a biofilm formation could be seen on the Ti surface, which was absent on all the other surfaces. This finding confirmed the improved antibacterial activity of the Zn-doped surfaces.

**Figure 10:**
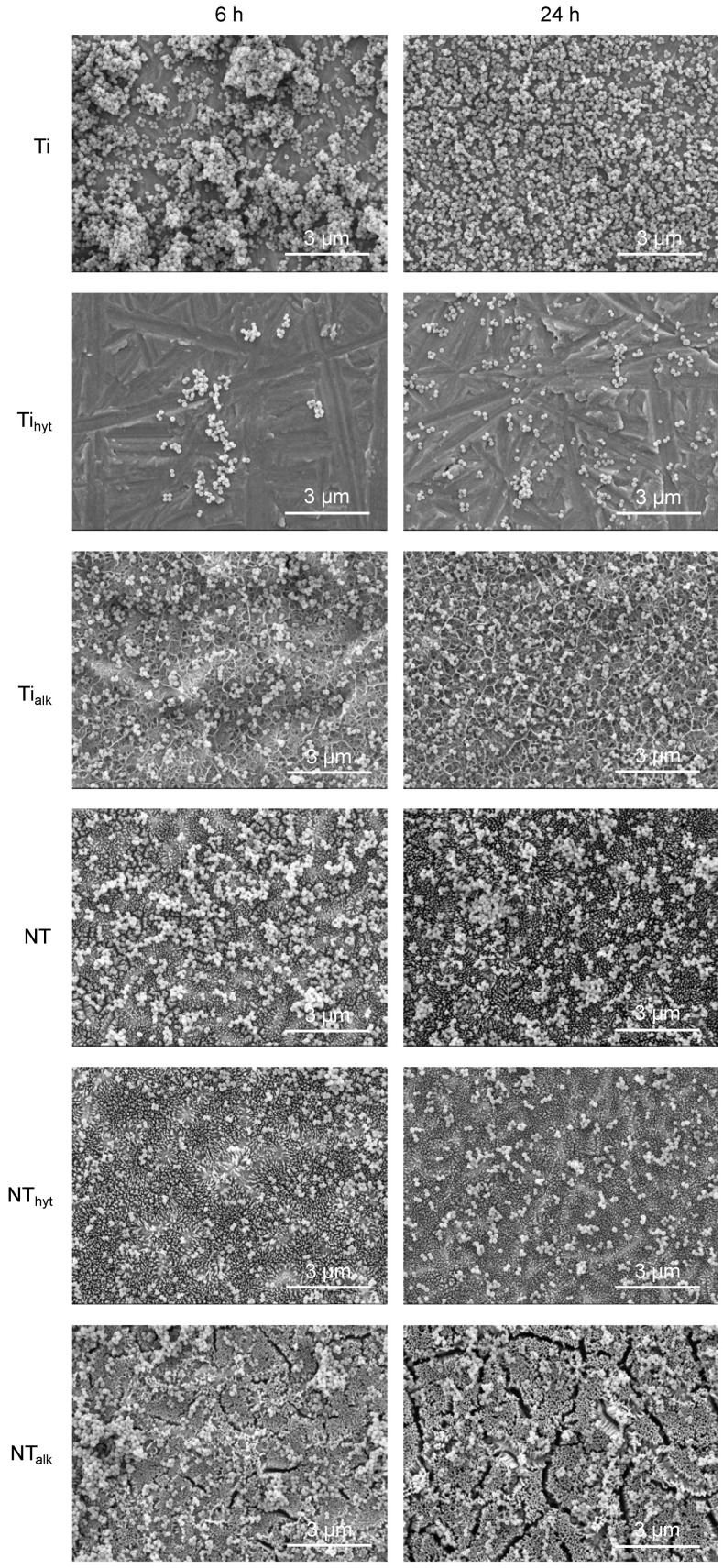
Representative SEM images of bacteria morphology after 6 h and 24 h of *S. aureus* incubation.

A similar result was observed when the surfaces were incubated with Gram-positive *S. aureus* (Figure 10). Biofilm formation was also observed on the Ti surface after 24 h of incubation with *S. aureus*, which was absent in all other surfaces. All the Zn-doped surfaces reduced the amount of *S. aureus* adhesion; however, the Ti_hyt_ and NT_hyt_ performed well in this case due to the higher amount of Zn presence on the surfaces. When compared to *P. aeruginosa*, the *S. aureus* adhesion was higher for all the surfaces. This is probably due to the surface charge difference and size of the two bacteria tested. Although bacteria have an electronegatively charged outer layer, they can differ from Gram-positive to Gram-negative because of the difference in cell-wall composition [54]. Since the TiO_2_ nanotube arrays have terminal hydroxyl groups which are negatively charged too, they repulse the bacteria and thus reduce bacteria adhesion on the NT surfaces [12]. For the differences in the electronegative charge between *P. aeruginosa* and *S. aureus*, the relative amount of antibacterial activity can differ, which was indicated in this case. Another important factor is the relative size of the two bacteria. *P. aeruginosa* is a rod-shaped bacterium having 1–5 µm of length and a 1 µm width [55]. On the other hand, *S. aureus* is a circular-shaped bacteria with a 1 µm diameter [56]. Due to this size difference, it is possible that the smaller *S. aureus* could evade the stretching force [12] induced by the NT arrays and resulted in more *S. aureus* attachment compared to *P. aeruginosa*.

## 4. Conclusions

Nanoscale features on Ti surfaces can improve complications such as bacterial infection and poor osseointegration. In this work, titania (TiO_2_) nanotube arrays (NT) were grown on pure Ti surfaces followed by zinc (Zn) doping. SEM images revealed noticeable morphological characteristics of the surfaces fabricated in this work. Hydrothermally treated Ti_hyt_ and NT_hyt_ surfaces had Zn crystals while the alkaline heat treatment created a web-like structure on the Ti_alk_ and NT_alk_ surfaces. EDS analysis indicated an even distribution of Zn on different surfaces. XPS confirmed the successful Zn doping on the surfaces while quantifying the amount of Zn. From the XPS, it was evident that hydrothermal treatment induced a higher amount of Zn on the surfaces. From the mechanical property analysis, it was found that the indentation hardness and elastic modulus of the nanotube surfaces (NT_hyt_ and NT_alk_) were significantly lower than the Ti surfaces. This indicates a suitable platform for cell attachment, growth, and proliferation. While testing with bacteria, all the Zn-doped surfaces exhibited significantly improved antibacterial characteristics compared to the pure Ti. These results signified the greater potential of nanoscale surface modification approaches to improve the biocompatibility of Ti-based orthopedic implants.

## Figures and Tables

**Table 1:** Elemental analysis (%at) of the surfaces from XPS survey spectra.

	% C	% O	% Ti	% Zn	% Na
Ti	55.6	34.7	9.7	0.0	0.0
Ti_hyt_	29.7	42.8	5.9	21.6	0.0
Ti_alk_	19.2	51.7	15.2	4.1	9.8
NT	21.3	57.3	21.4	0.0	0.0
NT_hyt_	17.7	53.6	11.7	16.9	0.0
NT_alk_	19.5	49.0	12.0	1.3	18.1

**Table 2:** %at of Zn and Na on the surfaces measured from XPS elemental analysis on Day 0 and Day 28. The negative values in the %at change column indicate increased concentration after 28 days.

	% Zn			% Na		
	Day 0	Day 28	% Change	Day 0	Day 28	% Change
Ti	0.0	0.0	0.0	0.0	0.0	0.0
Ti_hyt_	21.6	8.9	58.8	0.0	0.0	0.0
Ti_alk_	4.1	5.2	−26.8	9.8	4.4	55.1
NT	0.0	0.0	0.0	0.0	0.0	0.0
NT_hyt_	16.9	9.2	45.6	0.0	0.0	0.0
NT_alk_	1.3	2.6	−100.0	18.1	0.0	100.0

**Table 3:** Indentation hardness and elastic modulus values for different surfaces.

	Indentation Hardness (GPa)	Elastic Modulus (GPa)
Ti	2.30 ± 0.4	138 ± 20
Ti_hyt_	1.55 ± 0.08	94 ± 5
Ti_alk_	2.40 ± 0.2	91 ± 20
NT	0.32 ± 0.02	23 ± 1
NT_hyt_	0.26 ± 0.02	45 ± 4
NT_alk_	1.00 ± 0.4	76 ± 20

## Data Availability

Not applicable.
